# Significantly High Modulation Efficiency of Compact Graphene Modulator Based on Silicon Waveguide

**DOI:** 10.1038/s41598-018-19171-x

**Published:** 2018-01-17

**Authors:** Haowen Shu, Zhaotang Su, Le Huang, Zhennan Wu, Xingjun Wang, Zhiyong Zhang, Zhiping Zhou

**Affiliations:** 1 State Key Laboratory of Advanced Optical Communications System and Networks, Peking University, Beijing, 100871 China; 2 Key Laboratory for the Physics and Chemistry of Nanodevices, Peking University, Beijing, 100871 China

## Abstract

We theoretically and experimentally demonstrate a significantly large modulation efficiency of a compact graphene modulator based on a silicon waveguide using the electro refractive effect of graphene. The modulation modes of electro-absorption and electro-refractive can be switched with different applied voltages. A high extinction ratio of 25 dB is achieved in the electro-absorption modulation mode with a driving voltage range of 0 V to 1 V. For electro-refractive modulation, the driving voltage ranges from 1 V to 3 V with a 185-pm spectrum shift. The modulation efficiency of 1.29 V · mm with a 40-μm interaction length is two orders of magnitude higher than that of the first reported graphene phase modulator. The realisation of phase and intensity modulation with graphene based on a silicon waveguide heralds its potential application in optical communication and optical interconnection systems.

## Introduction

Silicon photonics has become one of the leading technological solutions for integrated photonics with its merits of low cost and CMOS compatibility. In particular, silicon modulators have been extensively studied as essential components of optoelectronic circuits^1–3^. A silicon modulator converts an applied external electrical signal into variations in the fundamental characteristics of light (including amplitude, phase, and polarisation) in an optical waveguide^4^. In general, there are two primary types of modulation, intensity modulation and phase modulation, which are suitable for short-reach interconnects and long-haul communication, respectively^5,6^. The purpose of the electro-optical modulator is to tune the light intensity and phase at the working wavelength. For a Mach-Zehnder optical modulator, the electro-optical effect of the modulation arms may result in the shifts of the transmission spectral, thus the change of the light intensity and phase would happen. The variation in light intensity make it possible for the generation of the amplitude-modulation signal. While the situation that the spectral shift with a *π* phase will be suitable for phase modulation^7^. For high-speed data transmission in integrated silicon photonic systems, the main approach to modulate optical signals is based on doped silicon p–n junctions^8^. However, this kind of modulator suffers from a large footprint, high energy consumption, and high insertion loss due to its relatively weak electro-refractivity, which requires millimetre device sizes to achieve a phase shift of *π*^1,9,10^. Modulation efficiency (*V*_*π*_ * *L*_*π*_) is the key parameter for evaluating performance of an electro-optical modulator, which describes the driving voltages and the device size required for the operational wavelength ideally drops to zero due to interference. The conventional carrier depletion based silicon optical modulators have a typical modulation efficiency of (5–30) V ⋅ mm^1,10,11^. In order to lift up the modulation efficiency, the other types of electro-optic materials have been assembled into the silicon waveguide, such as the metal-insulator-silicon modulator based on surface plasmonic polaritons (SPPs) and the organic-silicon hybrid (SOH) modulator, with the modulation efficiency coming up to 0.5 V ⋅ mm^12,13^.

Graphene, a typical 2D material with a single layer of carbon atoms, is emerging as a promising material for photonic applications owing to its unique optoelectronic propert ies^14^. Graphene-based devices hold great potential for enhancing the modulation efficiency with their advantages of compact footprint, low operation voltage, and ultrafast modulation speeds across broadband wavelengths. These properties of graphene have been exploited in electronic and photonic devices such as the electro-absorptive optical graphene modulator based on doped silicon waveguides^9,15–26^. An optical modulator with a graphene monolayer on a single silicon waveguide was first demonstrated in 2011^19^, and subsequently, a graphen,e modulator based on a micro-ring silicon waveguide was also exhibited, thereby demonstrating the potential of graphene integration with silicon waveguides^25^. Moreover, graphene modulator employing hybrid plasmonic effect was proposed for higher efficiency and more compact size^26^. However, the abovementioned graphene modulators are only based on the electro-absorption effect of graphene; these modulators may suffer a severe degradation in the signal-to-noise ratio when applied in long-haul communications. In order to meet the needs of phase-shift keying, Xu *et al*.^23^ have theoretically proposed a Mach–Zehnder (MZ) graphene modulator based on the doped silicon waveguide structure via analysing the electro-refractive effect of a graphene phase shifter, which suggests its potential in long-haul high-speed optical communication systems. In order to improve the modulation efficiency, few-layer graphene and novel waveguide structures in graphene based Mach-Zenhder phase modulator have also been discussed^27,28^. Furthermore, some other applications based on electro-refractive effect such as tunable polarizer has been demonstrated^29^. Recently, Mohsin *et al*.^9^ experimentally demonstrated electrorefractive graphene phase modulation by utilising a structure comprising double graphene layers. However, the device suffers from a large driving voltage range (−40–40) V and a relatively low modulation efficiency (300 V ⋅ mm).

In this study, we propose a graphene-based silicon MZ modulator which is switchable between the electro-absorption and electro-refractive modes of operation. Both simulations and experimental results show that the modulator affords the advantages of a low driving voltage, compact size, and high modulation efficiency. When the working range is (0–1) V, an extinction ration of 25 dB can be realised for electro-absorption modulation. For electro-refractive modulation, a maximum wavelength shift of 185 pm can be achieved within a driving voltage of (1–3) V. Further, the modulation efficiency is 1.29 V ⋅ mm with a 40-μm interaction length, which efficiency is two orders of magnitude higher than that of the first reported graphene phase modulator^9^.

## Results

### Concept and Principle

**Figure 1:**
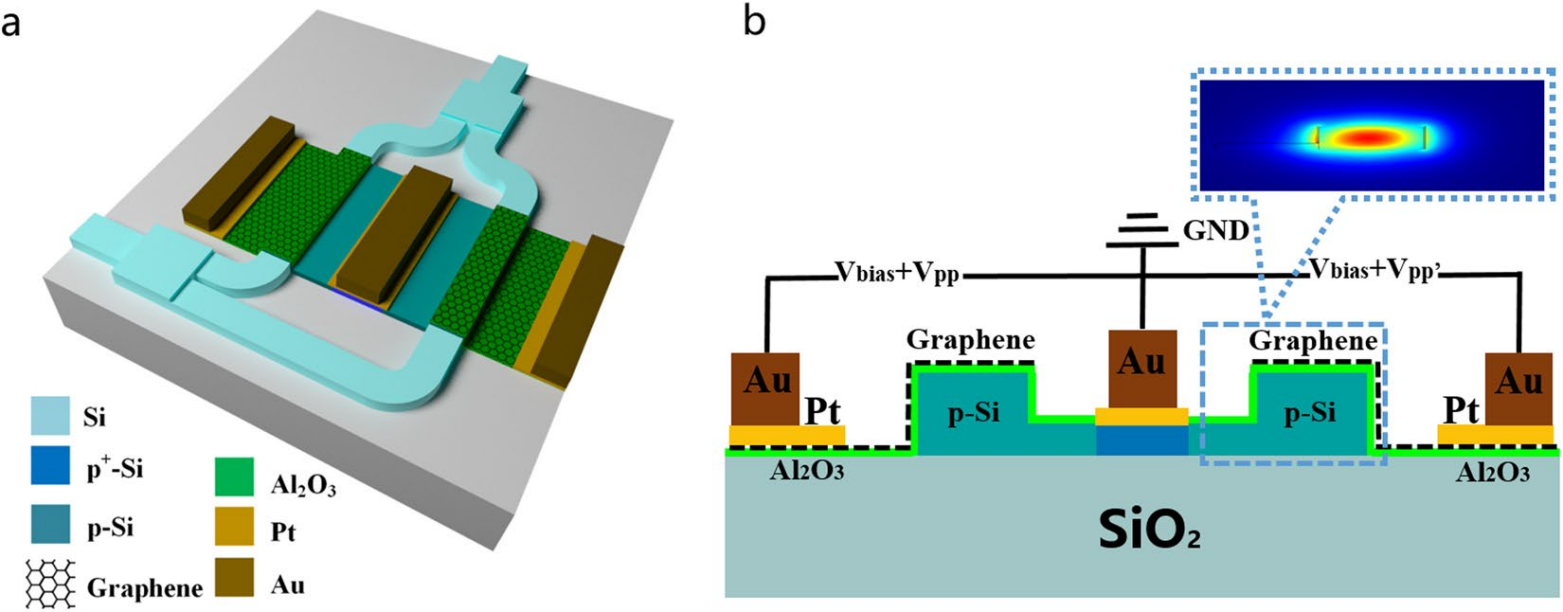
(**a**) Sketch of the Mach–Zehnder (MZ) modulator based on graphene-oxide-silicon (GOS) structure. (**b**) Cross-section of the interaction region with its drive scheme using a pair of differential signals. The inset shows the fundamental transverse electric (TE) mode distribution in one arm.

Figure 1(a) illustrates the sketch of the MZ modulator based on a graphene-oxide-silicon (GOS) structure, which is commonly used in optical communications and interconnection systems due to its wide bandwidth and temperature-insensitivity. The graphene layer is modelled as a thin layer between the oxide layer and the air cladding. In order to form a capacitor with graphene, the silicon can be p-doped to be electrically conductive. The alumina layer, acting as the dielectric of the capacitor structure between the graphene layer and doped waveguide, is used for adjusting the electric field of the evanescent wave in the silicon waveguide. Au-Pt electrodes are deposited on the graphene layer and the p-Si region to apply driving voltages or provide grounding. With change in the voltage applied to the graphene layer, the chemical potential (*μ*) of graphene can be tuned, as described by Eq. 1.

1 $$\mu =\hslash {v}_{F}\sqrt{\eta \pi |{V}_{g}-{V}_{{\rm{dirac}}}|}$$

where *v*_*F*_ = 3 × 10^6^ m ⋅ s^−1^ denotes the Fermi velocity, *V*_dirac_ = 0.8 V the voltage offset caused by natural doping, *V*_*g*_ the driving voltage, and *η* = 5.531 × 10^16^ m^−2^ ⋅ V^−1^ as per the GOS capacitor model.

The change in the chemical potential of the graphene layer results in variation in the optical conductivity of graphene, which can be described via Kubo formalism as below, owing to the contributions of the intraband and interband parts^9^.

2 $$\sigma (\omega ,{\mu }_{c},{\rm{\Gamma }},T)=\frac{j{e}^{2}(\omega -j2{\rm{\Gamma }})}{\pi {h}^{2}}\,[\frac{1}{{(\omega -j2{\rm{\Gamma }})}^{2}}{\int }_{0}^{+\infty }\xi (\frac{\partial {f}_{d}(\xi )}{\partial \xi }-\frac{\partial {f}_{d}(-\xi )}{\partial \xi })d\xi -{\int }_{0}^{+\infty }\frac{{f}_{d}(-\xi )-{f}_{d}(\xi )}{{(\omega -j2{\rm{\Gamma }})}^{2}-\mathrm{4(}\xi /h{)}^{2}}d\xi ]$$

Here, *ω*, *μ*_*c*_, *τ* and *T* represent the radian frequency, chemical potential, carrier relaxation time, and temperature. ${f}_{d}(\xi )={({e}^{(\xi -{\mu }_{c})/{k}_{B}T}+\mathrm{1)}}^{-1}$ is the Fermi-Dirac distribution, *ξ* is the energy, *k*_*B*_ is the Boltzmann constant. Accordingly, the relative permittivity of graphene changes with variation in the optical conductivity. Consequently, the effective refractive index (*n*_eff_) of the waveguide also changes, thus allowing phase shift realisation^25^. Figure 1(b) shows the differential drive scheme of the device. A pair of differential voltage signals based on the static working point with bias voltage is applied to the bottom graphene layer of the two arms. Consequently, a low driving voltage is achieved, which lowers the requirement of power consumption and intensity requirement of the frequency signal.

**Figure 2:**
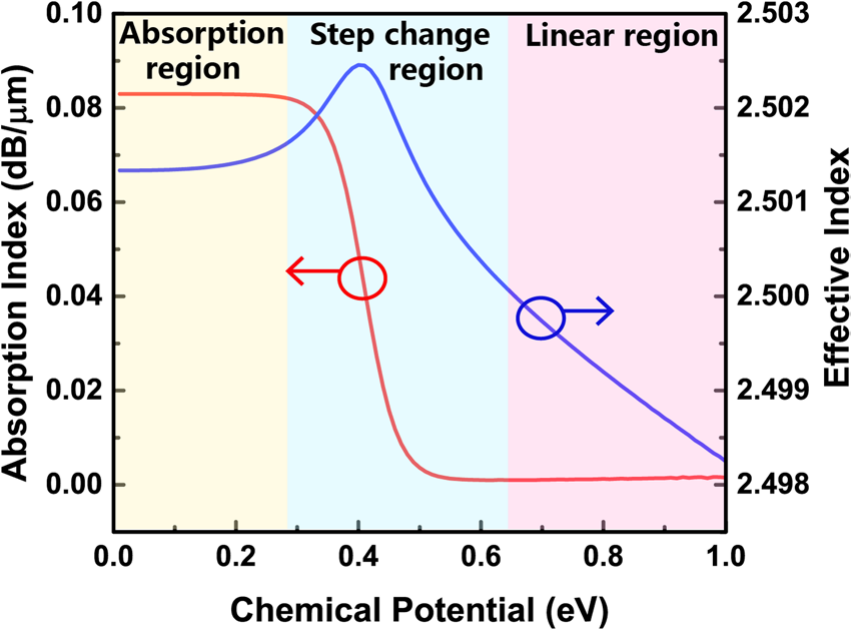
Simulation results of Re(*n*_eff_) and absorption ofgraphene-oxide-silicon (GOS) modulator as functions of the chemical potential.

We use COMSOL Multiphysics to analyse the mode distribution and photoelectric interaction in one arm of the MZ structure. Figure 2 shows the simulation results of Re(*n*_eff_) and the absorption of the GOS modulator for different chemical potentials. The fundamental transverse electric (TE) mode is solved around 2.5. As the chemical potential rises, Re(*n*_eff_) and the absorption coefficient exhibit three different sets of characteristics, which are classified in three stages. The range of (0–0.28) eV corresponds to the absorption region, wherein the absorption of GOS remains high and both the effective and absorption index change by very little. The step change region, (0.28–0.64) eV, is suitable for amplitude modulation, since there is a significant ‘stepping up’ of absorption index around 0.41 eV. In this region, the effective index of the TE fundamental mode first increases and subsequently decreases, wherein a peak value of *n*_eff_ can be observed around the 0.41 eV chemical potential point. Phase modulation schemes are applicable in the linear region, which lies in the range of (0.64–1) eV, as the absorption is low and the effective index exhibits a linear change.

### Fabrication

**Figure 3:**
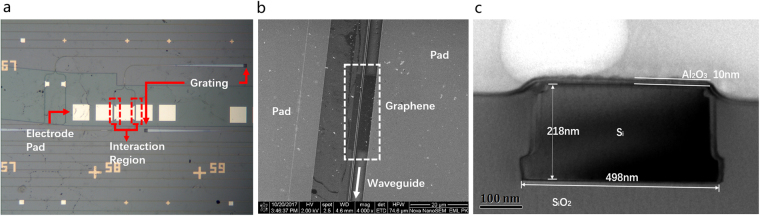
(**a**) Optical images of device (**b**) SEM planform image for interaction region (**c**) TEM image for the cross section of the graphene-oxide-silicon waveguide.

The proposed device is fabricated on a silicon-on-insulator (SOI) platform with ridge waveguides (width = 500 nm, height = 220 nm) on top of 2-μm buried oxide (BOX) and an interaction region with a length of 40 μm. Details of the fabrication process can be found in the Methods section. The optical images of the device are shown in Fig. 3(a). The device consists of three parts: electrode pads, interaction region, and grating couplers. The planform SEM image of the interaction region is shown in Fig. 3(b). The graphene layer could be observed clearly above the silicon waveguide with the electrical pads on the graphene layer, showing the good quality of the device fabrication. Figure 3(c) shows the TEM image of the waveguide cross-section. It is clear that the interface between silicon waveguide and the Al_2_O_3_ thin film can be recognized by the color depth of the different materials. The size of silicon waveguide is 498 nm × 218 nm, and the thickness of thin Al_2_O_3_ is 10 nm. It is the same with the size of our design.

### Simulation Results

**Figure 4:**
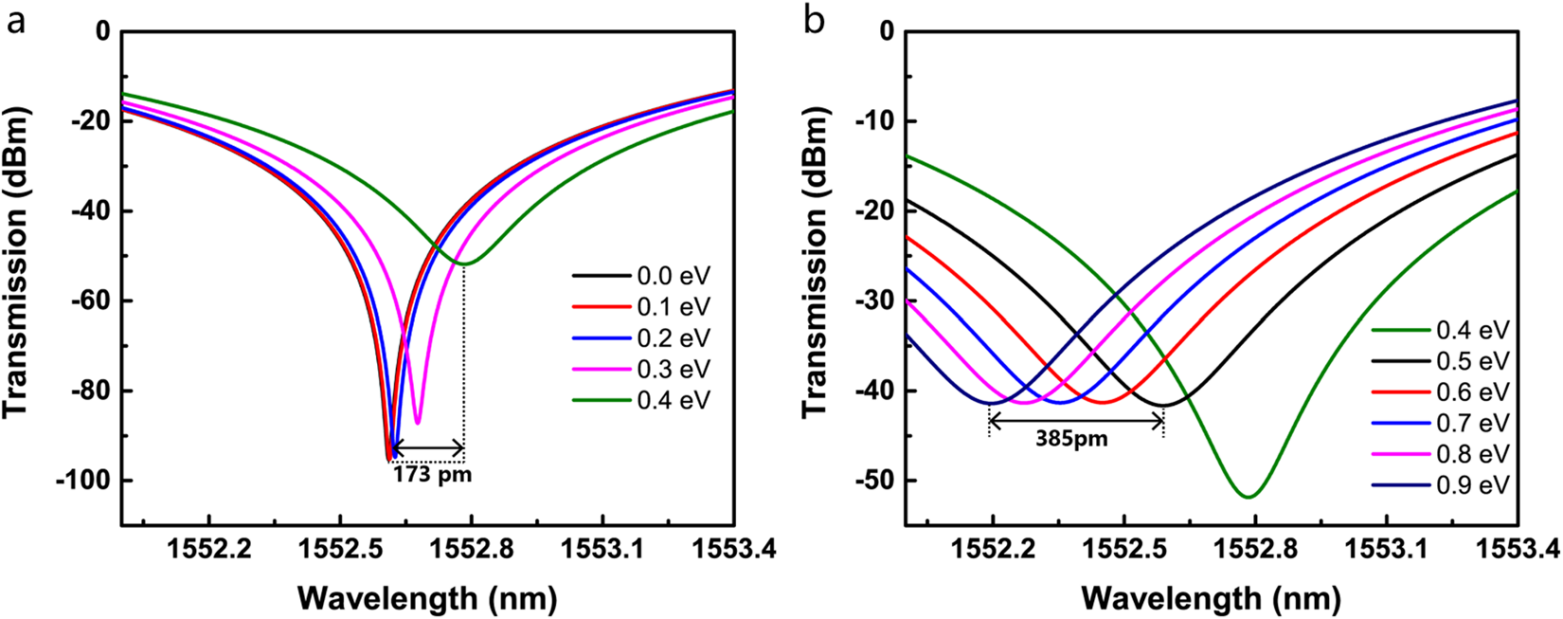
Transmission of modulator in (**a**) electro-absorption and (**b**) electro-refractive modes.

By applying different voltages to the GOS structure, the modulation mode can be switched to achieve either electro-absorption or electro-refractive. Here, the modulation-mode switch is mainly analysed by applying different voltages to a single arm. The simulation results of electro-absorption modulation and electro-refractive modulation are shown in Fig. 4(a) and (b), respectively.

When the chemical potential is in the range of (0–0.4) eV, corresponding to the absorption region and a part of the step region in Fig. 2, the device works as an amplitude modulator, with the centre wavelength around (1552.5–1552.8) nm. It is found that a large extinction ratio can be achieved in this operating region. A red-shift of about 173 pm is also observed. By tuning the voltage of the arm, the modulation mode can be switched to the phase-dependent mode when the chemical potential is in the range of 0.5 eV to 1 eV, corresponding to the linear region in Fig. 2. A 385-pm blue shift can be achieved in this range, with nearly no amplitude fluctuation. Thus, device operation in this region allows phase modulation.

### Experimental Results

**Figure 5:**
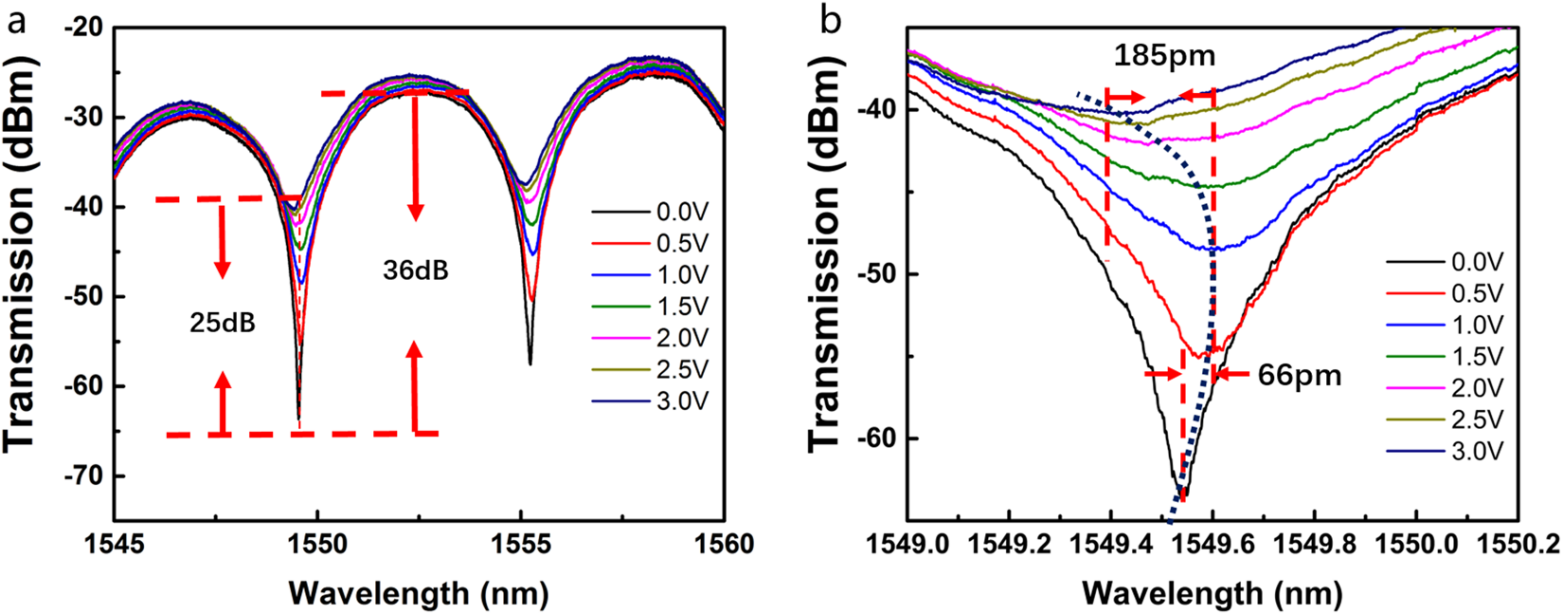
(**a**) Static spectra under different applied voltages. (**b**) Detailed spectra under different applied voltages.

Experiments have been designed to observe the modulation-mode switch of the device. Driving signal is applied to a single arm, which means *V*_pp_ keeps zero, *V*_bias_ equals to *V*_dirac_ (about 0.8 V) and *V*_pp′_ is changing from 0 V to 3 V. Figure 5(a) illustrates the transmission spectra for different value of *V*_pp′_. The static extinction ratio is 36 dB at 0 V, and it decreases with the increase in the applied voltage, which is attributed to the absorption of graphene. Such characteristics extend over a broad range of spectra and are not just limited to the range of (1545–1560) nm displayed in the figure. Figure 5(b) shows more details of the changes in the spectra near 1549.5 nm. In the voltage range of (0–1) V, the transmission spectra exhibit a relatively small red shift of about 66 pm, and there is a significant rise in the minimum value of light intensity. In the range of (1–3) V, the spectra exhibit a greater blue shift of about 185 pm and a significantly smaller change in the valley value, which confirms the modulator working in the phase-dependent mode. The experimental results validate the simulation results as regards the transmission of the modulator under different modulation modes (Fig. 4). According to the simulation, *n*_eff_ should increase till a chemical potential of 0.41 eV and begin to decrease subsequently, which corresponds to the blue and red shifts of the spectra, respectively.

## Discussion

**Figure 6:**
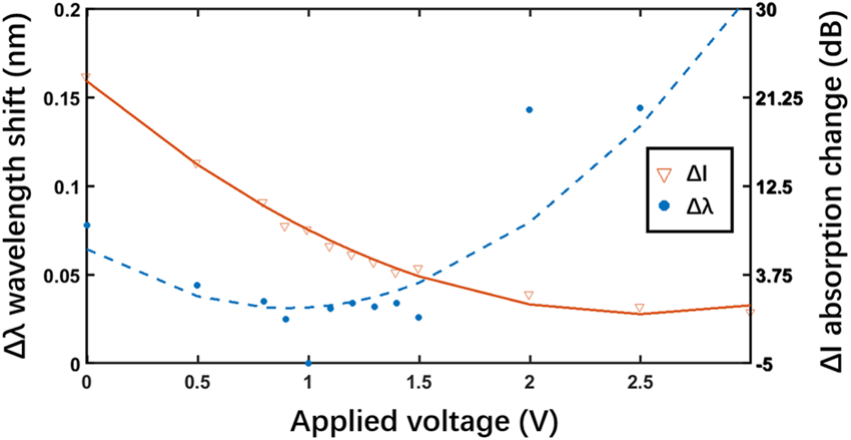
Spectra shift Δ*λ* and absorption variation Δ*l* for different applied voltages.

Figure 6(a) depicts the spectral shift (Δ*λ*) and absorption variation (Δ*l*) as functions of the applied voltage (for the voltage values shown in Fig. 5). The dots and triangles indicate the original data. The 1-V data point is set as the ‘0’ wavelength shift point and the transmission notch at 3 V is set as the ‘0’ absorption point. The phase and intensity variations from the ‘0’ point above are calculated for different applied voltages. We find from the fitting curves that the slope coefficient of Δ*l* is significantly larger than that of Δ*λ* in the region of (0–1) V, which means that intensity modulation is the preferred mode of operation. In contrast, the slope coefficient of Δ*λ* increases and dominates the region of (1–3) V, thereby indicating that phase modulation is more effective within this voltage range.

By measuring the shift of wavelength (Δ*λ*), we can obtain the refractive index change (Δ*n*) and phase shift change (*φ*) using Eqs 3 and 4, respectively, as below:

3 $${\rm{\Delta }}n(V)=\frac{\lambda }{L}[\frac{{\rm{\Delta }}\lambda (V)}{d}]$$

4 $${\phi }={\rm{\Delta }}{n}_{{\rm{eff}}}L\cdot \frac{2\pi }{\lambda }$$

here, Δ*λ*, *L* and *d* represent the wavelength shift, graphene length (40 μm), and free spectral range (6 nm), respectively. With application of the above equations, the observed wavelength shift of 185 pm in the voltage range of (1–3) V corresponds to a refractive index change of 0.0012 and phase shift change of 0.062*π*.

For on-chip phase modulators, the product of length L and drive voltage *V*_*π*_ for a phase shift of *π* is considered as a major figure^9^. From the above experimental results, we note that a 185-pm wavelength shift, translating into a phase shift of 0.062*π*, can be achieved for 2 V of applied voltage. This result indicates that the modulation efficiency *V*_*π*_ ⋅ *L*_*π*_ of the graphene modulator with 40-μm interaction region is 1.29 V ⋅ mm, which is an improvement of two orders of magnitude over the first reported electro-refractive graphene phase modulator whose modulation efficiency was 300 V ⋅ mm^9^. Further, our device offers the advantages of lower drive voltage, compact size, larger phase shift, and extinction ratio. The obtained high modulation efficiency is attributed to the ‘thinness’ of the alumina layer, which results in an applied voltage of only 2 V, and the high electrorefractive effect upon the strong interaction between the graphene and light within only the 40-μm length. The high modulation efficiency also validates the greater efficacy of the graphene electro-refractive effect over that of the conventional carrier depletion based silicon optical modulators with a typical value of (5–30) V ⋅ mm^1,10,11^.

Importantly, while our results validate the feasibility of using graphene, they also show exhibit good consistency with the theoretical results. Parameter Re(*n*_eff_) first increases and subsequently decreases as the chemical potential rises from 0 eV to 1 eV, with an inflection point at the chemical potential of 0.41 eV, corresponding to the two-stage wavelength shift and mode switch around 1 V observed in the experiments. Similarly, the absorption is relatively larger in the region of (0–0.41) eV but smaller in the region of (0.41–1) eV, corresponding to the different absorption changes over the voltage range of (0–1) V and (1–3) V in the experiments, which indicates that the chemical potential of 0.41 eV corresponds to the applied voltage of 1 V.

**Figure 7:**
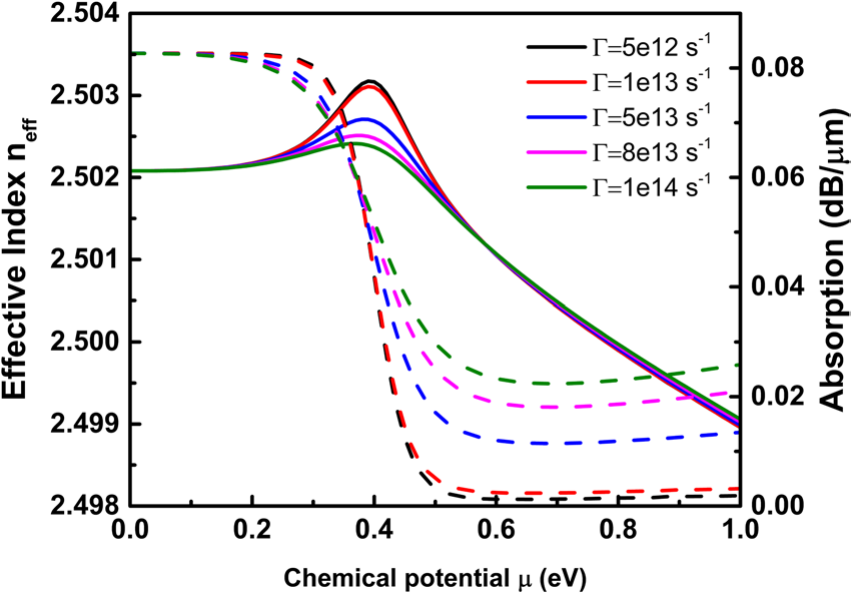
Simulated Re(*n*_eff_) and absorption of the GOS modulator under different scattering rate Γ.

However, it should be noted that the wavelength shifts observed in Fig. 5(a) are considerably smaller than those in simulation results. In both the red-shifted and blue-shifted regions, the simulation exhibits significantly larger wavelength shifts of 173 pm and 385 pm, respectively, compared with the shifts of 66 pm and 185 pm, respectively, observed in the experiments. Here, we speculate that such discrepency originates from the graphene mobility. The fabrication process determines the quality of the graphene sheet, hence affecting the mobility. Their effects on the optical conductivity are described by the scattering rate parameter Γ in Eq. 2. Typical value of the scattering rate in real graphene devices varies from 5e12 s^−1^ to 1e14 s^−1^, corresponding to charge carrier mobilities from 1500 to 30000 cm^2^/Vs at *μ* = 0.3 eV (calculated via ${{\rm{\mu }}}_{{\rm{m}}}={{\rm{ev}}}_{{\rm{f}}}^{{\rm{2}}}/({\rm{\Gamma }}{\rm{\mu }})$). The characteristics of the device under different scattering rates in this range are shown in Fig. 7. As the scattering effect increases, the bi-directional shift diminishes, especially for the red-shifted region. When Γ = 8e13 s^−1^, corresponding to mobility of 1875 cm^2^/Vs, the red shift reduces to 70 pm, close to the experimental measurements. To verify this assumption, a hall mobility measurement is performed on the same substrate with a graphene based hall element. The measuring method is the same as the previous work^30^. Our Hall mobility measurement performed on a similarly fabricated graphene yields 1650 cm^2^/Vs, which matches our theory well. We hereby consider the graphene mobility to be a major factor affecting the modulation performance in the real device.

It is suggested that the deviations of experiment and simulation in the extinction ratio measured in actual devices could be related to multiple factors. In the initial simulation, although the typical attenuation for silicon waveguide around 6 dB/cm loss has been taken into consideration, the MZI is assumed to have an ideal splitting ratio of 0.5:0.5. And the fabrication differences of the two arms were also neglected. Based on those assumptions, the simulation yielded a larger extinction ratio. However, several factors can greatly reduce the extinction ratio. The splitting ratio of the MMI in real fabrication and the loss difference in the two arms may result in an amplitude unbalance, which may cause the decrease of the extinction ratio.

**Figure 8:**
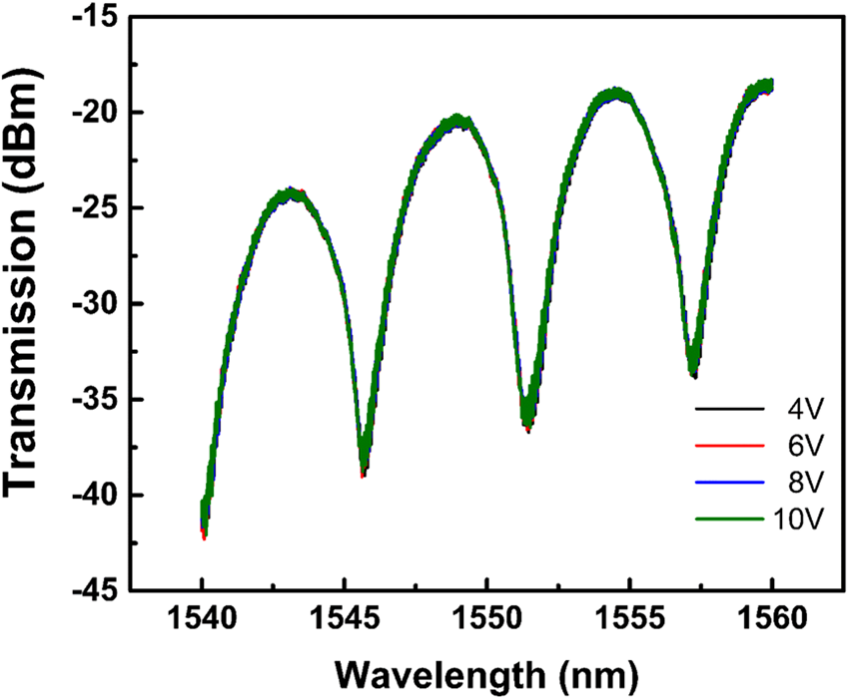
Static spectra for different applied voltages after a high voltage was applied to the device.

With increase in the applied voltage, small variations in the transmission spectra can be observed, but the spectra stop changing beyond a certain point. When applying the same set of voltages between 1 V to 3 V again to the same device, there is no wavelength shift anymore (see Fig. 8), which indicates a break-down in the device.

The behaviour of the device beyond the breakdown point also provides confirmation of our proposed modulation mechanism. In this situation, graphene breaks down while the plasma dispersion effect is still in process. However, no further spectral shift is observed anymore, which confirms that the phase shift mainly originates from the electro-refractive effect of graphene in the graphene-oxide-doped silicon capacitor structure. Although the plasma dispersion effect of the doped silicon still exists, considering that the doped region is relatively small and the plasma dispersion effect is weak in nature, the contribution of the doped silicon is negligible.

## Methods

### Fabrication

The graphene MZ phase modulator based on silicon waveguide device is realized on Si-on-insulator (SOI) platform with ridge waveguides (width = 500 nm, height = 220 nm) on top of 2 μm buried oxide (BOX). TE-polarized light was coupled in using grating couplers optimized for (1530–1570) nm. The relative difference between the lengths of two MZ arms is 91 μm. Subsequently, Al_2_O_3_ film with 10 nm thickness was deposited with atomic layer deposition (ALD) at 300 °C using H_2_O and trimethylaluminium (TMA) as precursors. The starting material for fabrication is the 8-inch (100) n-type SOI wafer. The SOI wafer was patterned by photolithography with an I-line and electron beam lithography (EBL) mixed. Patterns were then formatted using reactive ion etching (RIE). After the metal crossed shape marks (Ti/Au = 5/60 nm) were deposited for alignment of the following EBL processes. The graphene was grown on Pt film by CVD process and transferred onto the SOI platform by a bubbling method. Then, an EBL was carried out to form the graphene pattern, followed by a reactive ion etching (RIE) process to remove the unwanted graphene. Finally, another EBL was utilized to pattern the electrode pads, followed by an electron beam evaporation (EBE) process (Ti/Pd/Au = 0.5/30/50 nm) and a standard lift-off process.

### Measurement

A continuous-wave (CW) laser was employed to generate the optical signal. The polarization was set to TE by a deterministic polarization controller (DPC). The first EDFA after DPC was employed to ensure that enough power was input into the modulator. Different voltages were applied to the electrode of one arm. There was a second EDFA employed to amplify the modulated signal. An optical spectrum analyzer was employed to recieve the output signal with the center wavelength sweeping from 1545 nm to 1560 nm.

### Simulation

The proposed structures are simulated with 2D electromagnetic wave frequency-domain method provided by COMSOL Multiphysics. In this model, the graphene layer is represented as a thin (*d* = 0.7 nm) layer with anisotropic properties. The relative permittivity of graphene is described by a diagonal tensor. The in-plane component is *ε*_*xx*_ = *ε*_*zz*_ = 2.5 − (*jσ*/*ε*_0_*ωd*), while the surface-normal component *ε*_*yy*_ is set to 2.5. The offset of 2.5 on the relative permittivity is adopted from the relative permittivity of graphite. As long as the thickness of graphene layer *d* is sufficiently small, such as 1 nm or 0.3 nm, the simulation produces almost similar results.
